# Comparison between the CASIA SS-1000 and Pentacam in measuring corneal curvatures and corneal thickness maps

**DOI:** 10.1186/s12886-023-02768-w

**Published:** 2023-01-05

**Authors:** Robert M. Feldman, Gene Kim, Alice Z. Chuang, Atsushi Shiraishi, Keiichiro Okamoto, Makoto Tsukamoto

**Affiliations:** 1 Ruiz Department of Ophthalmology and Visual Science, McGovern Medical School at The University of Texas Health Science Center at Houston (UTHealth), 6400 Fannin St., Suite #1800, Houston, TX 77030 USA; 2 Department of Ophthalmology, Dell Medical School, Austin, TX USA; 3 Department of Ophthalmology, Ehime University Graduate School of Medicine, Shitsukawa, Toon, Ehime Japan; 4 Tomey Corp, Nagoya, Japan

**Keywords:** CASIA SS-1000, Pentacam, Corneal curvature, Laser refractive surgery

## Abstract

**Purpose:**

To compare the intra-device repeatability and inter-device reproducibility between two anterior segment imaging instruments, the CASIA SS-1000 (Tomey Corp., Nagoya, Japan) and Pentacam (OCULUS, Arlington, WA) in measuring anterior segment parameters.

**Methods:**

Single-center, prospective clinical trial. Participants ≥20 years of age were included. One eye was randomly selected, each imaged by three CASIA SS-1000 devices and three Pentacam devices by three different examiners. Each photographer operated a pair of devices, one CASIA SS-1000 and one Pentacam. The image order for each participant was determined by a random permutation table. Three images were taken from each device. A total of 18 images were taken for each eye. Ratios of the standard deviations, referenced as (CASIA/Pentacam), were calculated to compare the repeatability and reproducibility of the two imaging instruments.

**Results:**

In all, 66 participants with a mean age of 46.4 years (±21.7) were enrolled in the study. All repeatability ratios and intra-device variability were less than 1 (anterior corneal curvature: flat = 0.86, steep = 0.85; posterior corneal curvature: flat = 0.43, steep = 0.61; and map: thinnest = 0.22; central = 0.24, 2 mm = 0.26, 4 mm = 0.27, and 6 mm = 0.30). All reproducibility ratios, which measure the inter-device variability, were less than 1 (anterior corneal curvature: flat = 0.58, steep = 0.73; posterior corneal curvature: flat = 0.25, steep = 0.31; and pachymetry map: thinnest = 0.20; central = 0.20; 2 mm = 0.20; 4 mm = 0.19; and 6 mm = 0.22). A ratio of less than 1 indicates that the CASIA SS-1000 has more consistent measurements.

**Conclusions:**

The CASIA SS-1000 was found to have better repeatability and reproducibility compared to the Pentacam for both corneal curvature and pachymetry maps. This greater consistency may require further study to determine whether the decreased variability can be translated into improved clinical results.

## Introduction

Accurate measurements of the cornea are becoming increasingly important. Clinicians are using precise quantitative measurements of epithelium, stroma and endothelium to evaluate patients and improve surgical outcomes. Surgical planning before femtosecond laser-assisted treatments now requires precise stromal thickness to avoid sacrificing the stromal bed (< 250 μm). Pachymetry maps are relied on for any pre- and post-laser surgery evaluation, especially in subclinical and early diagnosis of corneal ectasia or form fruste keratoconus [1, 2]. Furthermore, morphological measurements, such as AC depth and angle, can prove vital in placing phakic intraocular lens (IOL) and recognizing complications of refractive surgeries [3].

Due to operator-dependent accuracy of contact tomography with ultrasound, accurate noncontact topography and tomography have evolved quickly. Scheimpflug systems, such as the Pentacam, allow for analysis of distorted and aberrated corneas, with true elevation measurements of both the anterior and posterior surfaces of the cornea. However, this technique is limited by cornea clarity, pupil diameter, and iris reflections [4]. Within the past decade, optical coherence tomography (OCT) imaging has increasingly been used to image the anterior chamber. Compared with Scheimpflug imaging, it boasts higher resolution and faster scans. With a newer swept-source OCT, such as the CASIA SS-1000, the increased frequency wavelength source decreases eye motion artifacts and scans deep corneal structures, due to little signal decay. The high-quality, cross-sectional images produce topographical maps of both the anterior and posterior corneal surfaces [5].

Since very small differences in corneal measurements alter clinical decisions, reproducibility and repeatability are extremely important in obtaining measurements from noncontact tomography. Studies have examined the inter-operator reproducibility and repeatability of a single Scheimpflug machine with a single OCT machine in corneal thickness and anterior chamber measurements in both healthy and postoperative eyes [6–11]. However, there has been no study looking at the inter-device reproducibility using multiple machines of the same type with the same operators. The purpose of this study is to compare both the intra-device repeatability and inter-device reproducibility between two anterior segment imaging instruments, the CASIA SS-1000 (OCT) and Pentacam (Scheimpflug imaging), in measuring anterior segment parameters.

## Participants and Methods

This prospective, single-center study was approved by the Institutional Review Board/Ethics at Ehime University Hospital, Shitsukawa, Toon, Ehime, Japan, and was registered on clinicaltrials.gov (NCT02077790) on 04/03/2014. All research adhered to the tenets of the Declaration of Helsinki and was HIPAA compliant.

### Participants

In all, 66 consecutive adult subjects (at least 20 years of age) were recruited. After obtaining informed consent, demographics, medical/ocular history, and present ocular medications were recorded. Eyes were excluded if they had any previous intraocular or corneal surgery (except laser refractive surgery), used a rigid contact lens within 1 week or soft contact lens within 24 hours, were currently on mydriatic and/or miotic medications, or were incapable of fixating at the internal fixation light or opening the eye sufficient to image a full image area. When both eyes of the participant were eligible, one eye was randomly selected by coin flip.

Study eyes underwent a series of imaging by three CASIA SS-1000 devices and three Pentacam devices. These devices were placed in three separate rooms. In each room, one CASIA SS-1000 and one Pentacam were installed and one examiner was assigned to operate the devices. Three images were taken by each of six devices. A computer-generated, randomization schedule was used to determine the order of the rooms/examiners and the order of the devices (CASIA SS-1000 vs. Pentacam) in a room. The exam procedure took approximately 1 h for imaging a total of 18 images in each eye. With this design, the device effect and examiner effect were confounded, and is called the [device+examiner] effect throughout the rest of this paper.

### Study instruments

#### CASIA SS-1000

The CASIA SS-1000 is a three-dimensional, noncontact, noninvasive, imaging system based on the principle of “swept-source” OCT. This system achieves high-resolution imaging of 10 μm (axial) and 30 μm (transverse) and high-speed scanning of 30,000 A-scans per second. All images were taken in 3D mode using the “corneal map” scan.

#### Pentacam

The Pentacam is a rotating Scheimpflug camera. It is a noncontact, noninvasive system that uses a monochromatic slit-light source (blue LED at 475 nm) for measuring anterior segment tomography. Twenty-five images with 500 measurement points on the front and the back of the corneal surface are acquired over a 180-degree rotation in 2 s. All images were taken by 3D scanning of 25 Picture/1 Sec*.* In addition, to reduce examiner-dependent variability, the CASIA SS-1000 auto-alignment/auto shot and Pentacam’s automatic release mode were used. This way each instrument automatically determines when correct focus and alignment is achieved and then performs a scan.

### Measurements

All measurements are performed in constant, dim-lighting conditions. Several corneal curvature and corneal thickness measurements were obtained from each image by each instrument:

1. Anterior axial power of the flattest meridian (AKf, D)
2. Anterior axial power of the steepest meridian (AKs, D)
3. Posterior axial power of the flattest meridian (PKf, D)
4. Posterior axial power of the steepest meridian (PKs, D)
5. Central corneal thickness (CCT, μm)
6. Thinnest corneal thickness (TCT, μm)
7. Horizontal location of thinnest corneal point (TCP(X, mm), in which 0 indicates the apex, positive value for the temporal direction, and negative value for the nasal direction (TCP(X), mm)
8. Vertical location of thinnest corneal point from apex (TCP(Y), mm), in which 0 indicates the apex, positive value for the superior direction, and negative value for the inferior direction
9. Peripheral corneal thickness at intersection between the 2-mm diameter circle and the nasal horizontal meridian (PCT2, μm)
10. Peripheral corneal thickness at intersection between the 4-mm diameter circle and the nasal horizontal meridian (PCT4, μm)
11. Peripheral corneal thickness at intersection between the 6-mm diameter circle and the nasal horizontal meridian (PCT6, μm)

### Data analysis

Demographics were summarized by mean and standard deviation (SD) for continuous variables or by frequency (%) for discrete variables. The repeatability and reproducibility were evaluated by:

- *Repeatability (intra-device) SD—*the variation of measurements taken by each device for each eye; and
- *Reproducibility (inter-device + intra-device) SD—*the variation of measurements taken by all three devices, by each instrument, for each eye.

Repeatability SD and Reproducibility SD were calculated for each device using a random effect model with random effects, participants (66 participants) and devices + examiners (three devices + examiners). The ratio of CASIA SS-1000 SD to Pentacam SD for each measure was computed for repeatability, reproducibility, and agreement. A ratio of less than 1 indicates that CASIA SS-1000 is more consistent than Pentacam, and vice versa. The coefficients of variation (CVs) were also calculated by repeatability SD/mean and reproducibility SD/mean.

Agreement, mean difference (bias) and limits of agreement (LOA) between CASIA SS-1000 and Pentacam was calculated. In addition, Deming regression analysis, which allows measurement errors in both CASIA SS-1000 and Pentacam, was used to examine any systematic deviations and estimate the conversion equations. Bland-Altman agreement plots and Deming regression plots were graphed.

Statistical analysis was performed using PROC MIXED procedure in SAS for Window 9.4 (SAS Inc., Cary, NC). Bland-Altman agreement plot was generated using BA.plot() and Deming regression analysis was performed using Deming() in MethComp package using R × 64 Version 3.01.

## Results

In all, 66 eyes of 66 participants were included in the study. The mean (±SD) age was 46 (±22) years (range 23–79 years). Of 66 study eyes, 32 (48%) were right eyes and 35 (53%) were from male subjects. Most eyes had no corneal pathology (54, 82%). Other ocular pathologies can be seen in Table 1.

**Table 1:** Demographics and Ocular Characteristics

Variable	ALL (***N*** = 66)
Age (year, mean ± SD [Range])	46.4 ± 21.7 [23 to 79]
Sex (male, %)	35 (53%)
Race (Asian, %)	66 (100%)
Study eye (Right, %)	32 (48%)
Corneal Pathology (%)	
None	54 (81.8%)
Dry eye	4 (6.1%)
Cataract	3 (4.6%)
Fuchs dystrophy	2 (3.0%)
Previous refractive surgery	1 (1.5%)
Sjogren syndrome	1 (1.5%)
Herpes keratitis	1 (1.5%)

### Intra-device repeatability

Complete results are shown in Table 2. All repeatability SD ratios for corneal curvature and thickness measurements were less than 1, which indicates that the CASIA SS-1000 is more consistent within an eye measured by the same device and the same operator, especially the corneal thickness measures (0.22–0.30). The repeatability of anterior corneal curvature between the two instruments were similar (ratio = 0.86 and 0.85 for anterior flat and steep meridian curvature, respectively). All coefficients of variation (CV) (SD/mean) for corneal curvature and thickness measurements were less than 0.5% for CASIA SS-1000 and 1% for Pentacam. The repeatability for TCP(X) was similar between two instruments (SD ratio = 1.06 with CV = 48.8% for CASIA; 54.3% for Pentacam), and CASIA was more repeatable in TCP(Y) than Pentacam (SD ratio = 0.68 with CV = 35.6% for CASIA and 60.5% for Pentacam).

**Table 2:** Repeatability and Reproducibility

Parameter	Mean			Inter-device		Repeatability (Intra-device)			Reproducibility (Intra-device + Inter-device)			Repeatability CV (%)		Reproducibility CV (%)	
	CASIA	Pent	P	CASIA (SD)	Pent (SD)	CASIA (SD)	Pent (SD)	Ratio	CASIA (SD)	Pent (SD)	Ratio	CASIA	Pent	CASIA	Pent
**AKf (D)**	43.29	43.29	0.71	0.06	0.21	0.14	0.17	**0.86**	0.16	0.27	**0.58**	0.3	0.4	0.4	0.6
**AKs (D)**	44.17	44.18	0.39	0.10	0.17	0.16	0.18	**0.85**	0.18	0.25	**0.73**	0.4	0.4	0.4	0.6
**PKf (D)**	51.01	52.14	< 0.001	0.06	0.57	0.16	0.38	**0.43**	0.17	0.69	**0.25**	0.3	0.7	0.3	1.3
**PKs (D)**	53.52	54.86	< 0.001	0.10	0.55	0.25	0.41	**0.61**	0.27	0.69	**0.39**	0.5	0.8	0.5	1.3
**TCT (μm)**	527.48	538.71	< 0.001	2.02	10.07	1.06	4.91	**0.22**	2.28	11.2	**0.20**	0.2	0.9	0.4	2.1
**TCP(X) (mm)**	0.41	0.35	0.001	0.06	0.13	0.20	0.19	**1.06**	0.21	0.23	**0.92**	48.8	54.3	51.2	65.7
**TCP(Y) (mm)**	−0.45	−0.38	0.004	0.07	0.24	0.16	0.23	**0.68**	0.17	0.33	**0.52**	35.6	60.5	37.8	86.8
**CCT (**μm**)**	533.68	544.13	< 0.001	1.83	9.70	1.03	4.26	**0.24**	2.10	10.59	**0.20**	0.2	0.8	0.4	1.9
**PCT2 (μm)**	543.69	554.96	< 0.001	1.93	10.26	1.23	4.69	**0.26**	2.29	11.28	**0.20**	0.2	0.8	0.4	2.0
**PCT4 (μm)**	565.68	582.89	< 0.001	2.00	11.60	1.44	5.32	**0.27**	2.46	12.76	**0.19**	0.3	0.9	0.4	2.2
**PCT6 (μm)**	602.03	632.55	< 0.001	2.36	12.51	1.96	6.62	**0.30**	3.07	14.16	**0.22**	0.3	1.0	0.5	2.2

*SD* Standard deviation, *AKf* Anterior curvature flat axis, *AKs* Anterior curvature steep axis, *PKf* Posterior curvature flat axis, *PKs* Posterior curvature steep axis, *TCT* Thinnest corneal thickness, *TCP(X)* Horizontal distance from the apex to the thinnest corneal point with positive value for the temporal direction and negative value for the nasal direction, *TCP(Y)* Vertical distance from the apex to the thinnest corneal point, *CCT* Central corneal thickness, *PCT* Peripheral corneal thickness, *Pent* Pentacam

### Inter-device reproducibility

All reproducibility SD ratios were less than 1, indicating the CASIA SS-1000 is more consistent (Table 2). The ratios for corneal thickness ranged from 0.19–0.22, indicating the Pentacam’s inter-[device+examiner] variation was 5 times greater than the CASIA SS-1000. The ratios for posterior corneal curvature ranged from 0.25–0.39. All CVs for corneal thickness and curvature measurements were less than 0.5% for CASIA SS-1000 and 2.2% for Pentacam. The reproducibility of TCP(X) was similar between the two instruments (SD ratio = 0.92 and with CV = 51.2% for CASIA and 65.7% for Pentacam), while CASIA had a better reproducibility in TCP(Y) (SD ratio = 0.52 with CV = 37.8% for CASIA and 86.8% for Pentacam).

### Agreement

**Fig. 1:**
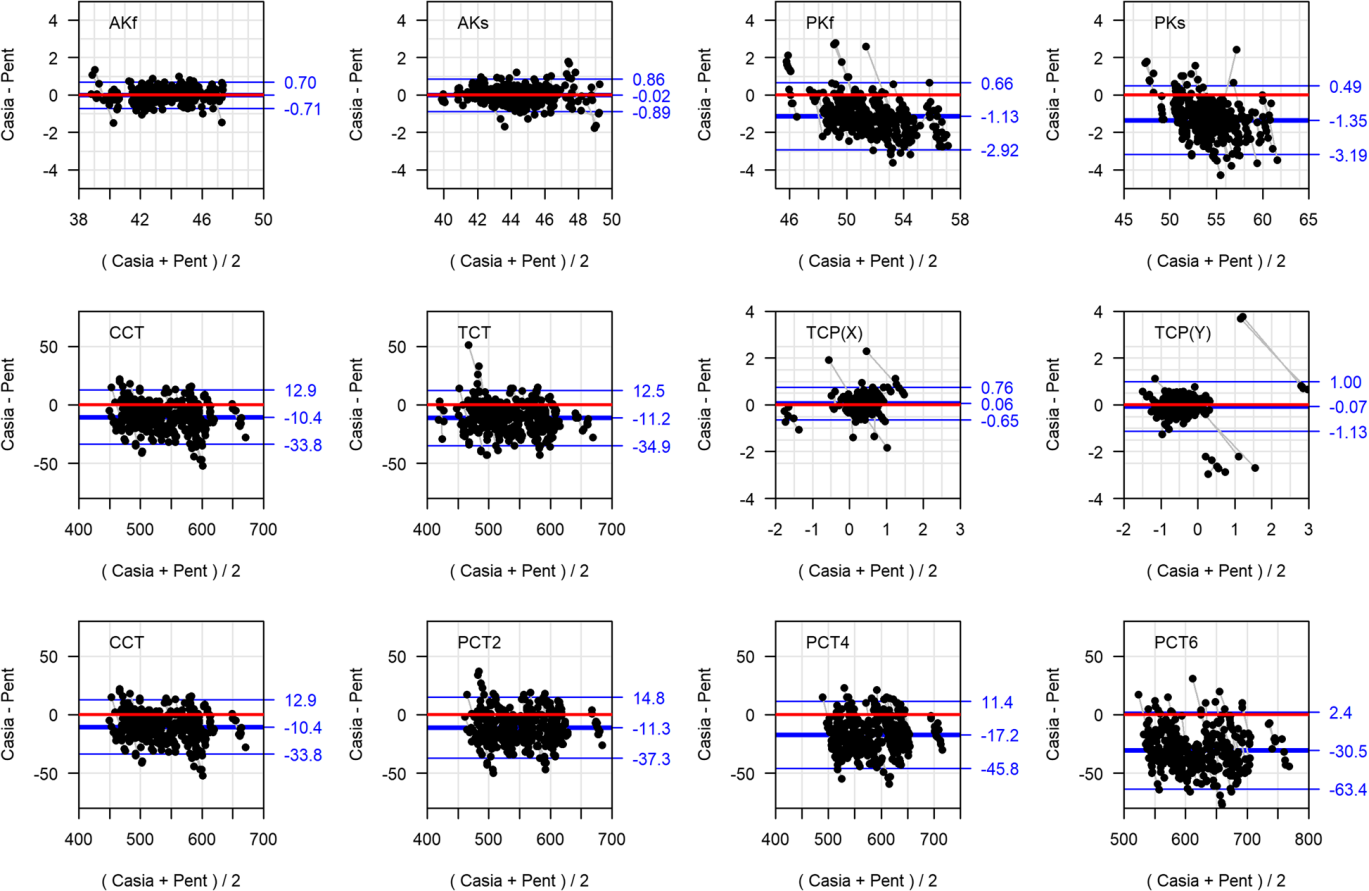
Bland-Altman plots: Red line indicate CASIA SS-1000 and Pentacam are identical (difference = 0); Blue lines = limits of agreement (LOAs); Black dots connected with grey lines are the measurements from the same subject. AKf = anterior curvature flat axis; AKs = anterior curvature steep axis; PKf = posterior curvature flat axis; PKs = posterior curvature steep axis; TCT = thinnest corneal thickness; TCP(X) = horizontal distance from the apex to the thinnest corneal point; TCP(Y) = vertical distance from the apex to the thinnest corneal point; CCT = central corneal thickness; PCT = peripheral corneal thickness

**Fig. 2:**
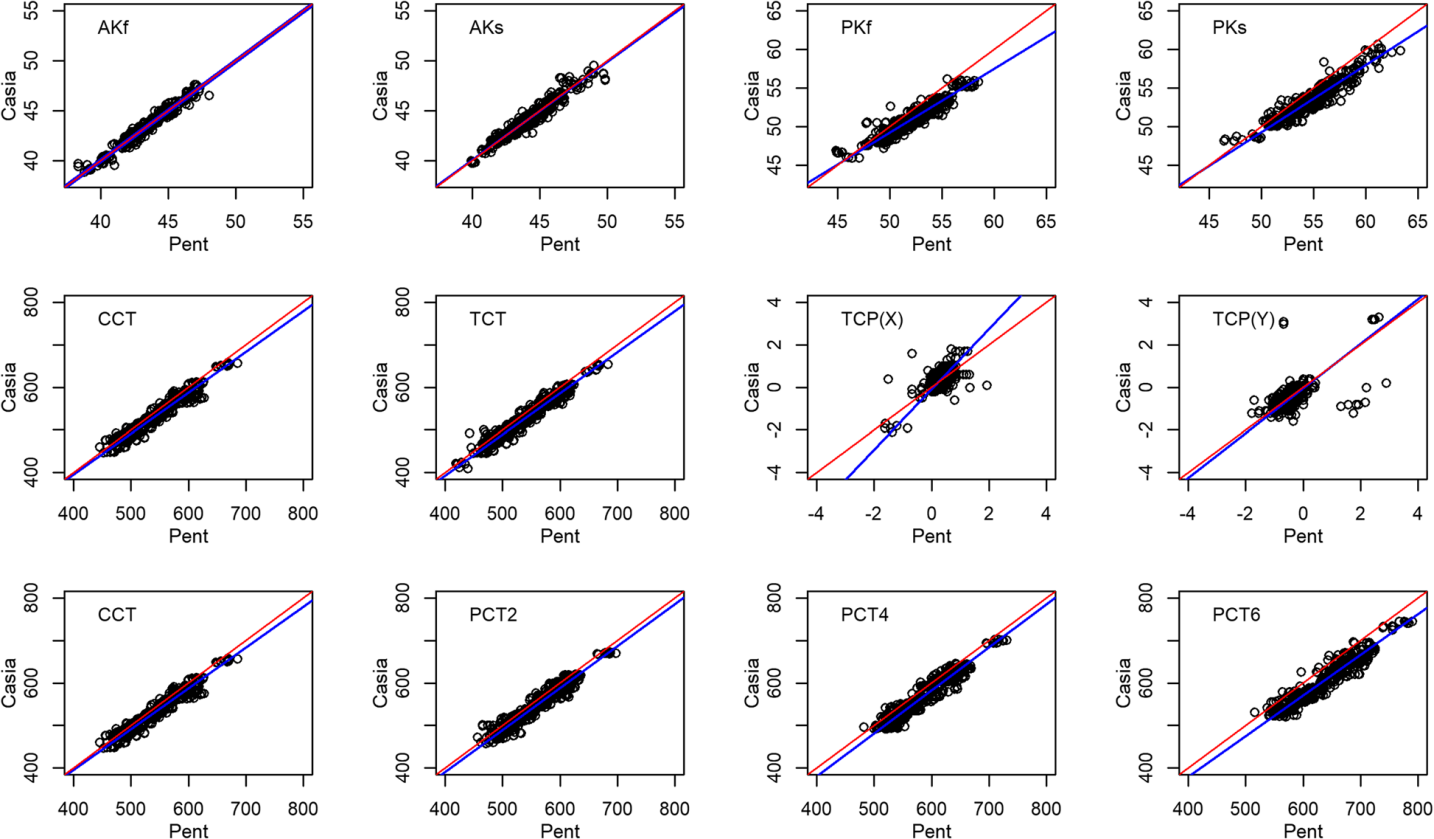
Deming Regression plots: Red line indicate CASIA SS-1000 = Pentacam; Blue line = Deming regression line; Black circles are the measurements. AKf = anterior curvature flat axis; AKs = anterior curvature steep axis; PKf = posterior curvature flat axis; PKs = posterior curvature steep axis; TCT = thinnest corneal thickness; TCP(X) = horizontal distance from the apex to the thinnest corneal point; TCP(Y) = vertical distance from the apex to the thinnest corneal point CCT = central corneal thickness; PCT = peripheral corneal thickness

Bland-Altman agreement plots and Deming regression plots are shown in Figs. 1 and 2, respectively. Anterior corneal curvature had excellent agreement between CASIA SS-1000 and Pentacam. The mean difference between the two instruments (see Table 2 and Fig. 1) was 0.01 D (*P* = 0.71) and 0.02 D (*P* = 0.39) for flat and steep meridian, respectively, and 95% of the differences for the flat meridian were within 0.70 D and 0.87 D for the steep meridian. The CASIA SS-1000 posterior corneal curvature was flatter by 1.13 D (*P* < 0.001) and 1.35 D (*P* < 0.001) in flat and steep meridian, respectively, compared to the Pentacam. The widths of LOAs for posterior corneal curvatures were about 2.5 times wider than the LOA widths for anterior curvatures.

Corneal thickness measured by CASIA SS-1000 was thinner compared with the values obtained with the Pentacam in various locations. The mean difference in TCT between CASIA SS-1000 and Pentacam was − 11.20 μm (*P* < 0.001) with LOA of [− 34.9 12.5]. The mean difference in CCT between CASIA SS-1000 and Pentacam was − 10.45 μm (*P* < 0.001) with LOA of [− 33.75, 12.85]. The differences and LOA increased when the location of measurements was away from the central cornea (Fig. 1). The mean difference in TCP(X) and TCP(Y) between CASIA SS-1000 and Pentacam was 0.06 mm (*P* = 0.001) and − 0.07 mm (*P* = 0.004), respectively, and 95% of the differences for TCP(X) were within 0.70 mm, which was smaller than 1.07 mm for TCP(Y) (Fig. 1). Further, Bland-Altman plots showed no special distribution patterns, such as significant trend in mean difference or varying the LOA width.

Deming regression plots (Fig. 2) showed that the all slopes of Deming regression lines (blue lines) were parallel to the red lines (CASIA SS-1000 = Pentacam or slope = 1), except flat and steep posterior corneal curvatures and TCP(X). The slope of flat and steep was 0.83 and 0.87, respectively, which indicates that CASIA SS-1000 measurements are smaller than Pentacam when the posterior curvature increases. The slope of TCP(X) was 1.42 and intercepted with the line with slope = 1 around the apex (TCP(X) = 0), which implies that CASIA SS-1000 measured farther away from the apex than Pentacam. However, the difference may not be clinically significant. A linear conversion between measurements, taken by CASIA SS-1000 and Pentacam, was derived from Deming regression analysis (Table 3).

**Table 3:** Conversion equation between CASIA SS-1000 and Pentacam from Deming Regression Analysis

Measurement	Conversion Equation by Deming Regression Equation
AKf (D)	CASIA = 0.032 + 0.999 x Pentacam
AKs (D)	CASIA = 0.717 + 0.983 x Pentacam
PKf (D)	CASIA = 7.905 + 0.827 x Pentacam
PKs (D)	CASIA = 5.758 + 0.870 x Pentacam
TCT (μm)	CASIA = 6.88 + 0.97 x Pentacam
TCP(X) (mm)	CASIA = -0.092 + 1.417 x Pentacam
TCP(Y) (mm)	CASIA = -0.048 + 1.051 x Pentacam
CCT (μm)	CASIA = 10.87 + 0.96 x Pentacam
PCT2 (μm)	CASIA = −4.63 + 0.99 x Pentacam
PCT4 (μm)	CASIA = −25.58 + 1.01 x Pentacam
PCT6 (μm)	CASIA = −3.96 + 0.96 x Pentacam

*AKf* Anterior curvature flat axis, *AKs* Anterior curvature steep axis, *PKf* Posterior curvature flat axis, *PKs* Posterior curvature steep axis, *TCT* Thinnest corneal thickness, *TCP(X)* Horizontal distance from the apex to the thinnest corneal point with positive value for the temporal direction and negative value for the nasal direction, *TCP(Y)* Vertical distance from the apex to the thinnest corneal point, *CCT* Central corneal thickness, *PCT* Peripheral corneal thickness

## Discussion

This study aims to evaluate the repeatability, reproducibility and agreement of the CASIA SS-1000 and Pentacam in measuring the anterior segment parameters in an eye, including corneal curvature and corneal thickness maps. Of the eight parameters studied, flat and steep meridians of the anterior corneal curvature (AKf and AKs) and CCT/TCT are the most important parameters in making clinical decisions.

### Anterior corneal curvatures

Anterior curvatures, along with axial length, are the key factors in calculating IOL power. A difference of more than 1 D in anterior corneal curvature is considered as clinically significant. The repeatability and reproducibility were excellent (SDs < 0.3 D). However, the CASIA SS-1000 had a better reproducibility in measuring flat axis (ratio = 0.58). The average difference in AKf and AKs between the two devices was 0.01 D and − 0.02 D, respectively. LOA limits are within 1 D. Thus, the two instruments are substantially equivalent, however, the CASIA SS-1000 had more consistent results.

### Thinnest and central corneal thickness

TCT and CCT are important parameters for preoperative refractive surgery screening. In refractive surgery, based on the Munnerlyn formula [12], every 12 μm of ablation depth will correct 1 D of the spherical equivalent for a single 6 mm ablation zone. Depending on the lasers and ablation zones, the average corneal ablation depth ranged from 12 to 17 μm per diopter [12]. In addition, sufficient thickness of both flap and residual posterior stroma is required to prevent irregular astigmatism and to avoid diseases of corneal integrity and/or postoperative corneal ectasia. In general, a minimum of 110 μm flap will be created, leaving a minimum of 250 μm of residual posterior stroma. Thus, the accuracy of TCT and CCT are extremely important for thin corneas and high-myopia corrections, which require greater ablation depth. An overestimate of TCT and CCT during refractive surgery screening may result in postoperative complications. Therefore, an overestimate of 12–17 μm is considered clinically significant.

CASIA SS-1000 is 4x (repeatability ratio = 0.22–0.24) more precise than Pentacam when repeating TCT and CCT measurements using the same device, and 5x (repeatability ratio = 0.20) more precise than Pentacam when repeating TCT and CCT measurements using different devices.

The mean difference in CCT between CASIA SS-1000 and Pentacam of − 10.45 μm (~ 2% of average CCT for all participants) with LOA ranging 45 μm (− 33.75, 12.85) could be considered clinically significant. This difference may lead ophthalmologists to make a safer (more conservative) decision for refractive surgery. However, it may disqualify some patients who would be qualified if measured by Pentacam. The two measurements can be converted by the following equation: CASIA = 10.87 + 0.97 x Pentacam. For example, an eye measured 500 μm CCT by Pentacam can be converted to CASIA measurement as 490.87 μm. Therefore, decreasing the clinical difference in the values can be obtained. Similarly, TCT measurements can be converted by CASIA = 6.88 + 0.97 Pentacam. However, the decreased variability in measurements obtained with the CASIA SS-1000 results in more consistent clinical decisions.

In addition, after reviewing 94 papers published between 2000 and 2014, Rozema et al. reported that of the 14 instruments in which Pentacam was compared, five significantly overestimated CCT in normal eyes [13]. These included TMS-5 (Tomey Corp, Nagoya, Japan, by 17 μm), Orbscan with AF (Bausch & Lomb, Claremont, CA, by 11 *μ*m), Visante/Stratus (Carl Zeiss, Dublin, CA, by 14 *μ*m), SL-OCT (Heidelberg Engineering, Heidelberg, Germany, by 16 *μ*m), and specular microscopes (by 20 *μ*m). Other studies showed similar results, with Pentacam overestimating by an average of 17 *μ*m, compared to Visante OMNI (Carl Zeiss, Dublin, CA) (17.8 ± 11.3 μm) [14], and Lenstar (Haag-Streit AG, Koeniz, Switzerland) (17.1 ± 8.5 *μ*m) [15].

### Posterior corneal curvature

Recent studies have shown that posterior corneal curvature, especially maximal point of elevation over the best-fit reference image, was statistically significant for keratoconus [16]. The ability to detect subclinical keratoconus earlier has important implications for improving refractive surgical screening to ensure that patients do not develop subsequent ectasia [16]. In theory, flat and steep posterior corneal curvature (PKf and PKs) may provide useful information for more accurate toric IOL calculations. However, measured vs. assumed posterior corneal curvatures have shown no difference in IOL calculation accuracy [17]. Our study showed that the CASIA SS-1000 underestimated the posterior corneal curvature comparing to the Pentacam. It is difficult to know what implications that an underestimate will have on screening for early keratoconus. The topographic morphologic distribution of the posterior corneal surface, specifically looking at the superior and inferior asymmetry within one topographic image, is a better early indicator of subclinical keratoconus than the raw absolute value of aggregate posterior corneal curvature being higher. Further studies are needed to analyze the topographic map of the posterior surface—and not just a solitary absolute value of the mean corneal curvature.

The absolute value of the posterior surface being smaller will affect the IOL calculation if the posterior corneal curvature value is used to calculate the total corneal power. However, recent studies using the Barret toric calculator showed that an estimated posterior curvature is just as accurate as a measured corneal curvature when looking at post-cataract refractive outcomes. As the Barrett formula is the current standard for toric IOL calculations, the difference in absolute value measurement of the posterior corneal surface between the Pentacam and CASIA will have little clinical difference in accuracy when correcting astigmatism, as the assumption has shown equivalent results to measured values. The anterior corneal surface measurements between the Pentacam and CASIA, which are more important in corneal measurement when performing IOL calculations, were not statistically different.

### Peripheral corneal thickness

The corneal thickness and location data has less variability for the CASIA, compared to the Pentacam, in both repeatability and reproducibility. The CASIA corneal thickness measurements are also consistently lower in value than the Pentacam corneal thickness measurement in every point in the periphery: PCT2, PCT4, and PCT6. The pattern/distribution of corneal thickness (pachymetry map) and the location of the thinnest point in the corneal is important in planning for refractive surgery and detecting corneal diseases, such as keratoconus [18]. Thus, less variability in both the corneal thickness measurements and location of thinnest corneal point are all very important clinical measurements for screening laser refractive surgery patients.

## Conclusion

This paper is the first to compare the inter-user repeatability and inter-device reproducibility of the Pentacam and CASIA SS-1000. The instruments were found to be substantially equivalent for planning IOL implantation surgery. Measurements in corneal thickness, although not identical, can be adjusted by the formula reported herein. Additionally, the variability is considerably less with the CASIA SS-1000 and, thus, offers more consistent results, an important factor in laser refractive surgery.

## Data Availability

The datasets used and/or analysed during the current study are available from the corresponding author on reasonable request. The authors do not wish to share the dataset in a public repository due to ongoing regulatory review.

## Abbreviations

AKf: Anterior curvature flat axis
AKs: Anterior curvature steep axis
CCT: Central corneal thickness
CV: Coefficient of variation
IOL: Intraocular lens
LOA: Limits of agreement
OCT: Optical coherence tomography
PCT: Peripheral corneal thickness
PKf: Posterior curvature flat axis
PKs: Posterior curvature steep axis
SD: Standard deviation
TCP(X): Horizontal distance from the apex to the thinnest corneal point
TCP(Y): Vertical distance from the apex to the thinnest corneal point
TCT: Thinnest corneal thickness
